# Mitochondrial Dysfunction, Oxidative Stress and Neuroinflammation in Neurodegeneration with Brain Iron Accumulation (NBIA)

**DOI:** 10.3390/antiox9101020

**Published:** 2020-10-20

**Authors:** Isabel Hinarejos, Candela Machuca, Paula Sancho, Carmen Espinós

**Affiliations:** 1 Unit of Genetics and Genomics of Neuromuscular and Neurodegenerative Disorders, Centro de Investigación Príncipe Felipe (CIPF), 46012 Valencia, Spain; mihinarejos@cipf.es (I.H.); cmachuca@cipf.es (C.M.); psancho@cipf.es (P.S.); 2 Rare Diseases Joint Units, CIPF-IIS La Fe & INCLIVA, 46012 Valencia, Spain; 3 Unit of Stem Cells Therapies in Neurodegenerative Diseases, Centro de Investigación Príncipe Felipe (CIPF), 46012 Valencia, Spain; 4 Department of Genetics, University of Valencia, 46100 Valencia, Spain

**Keywords:** neurodegenerative disorder, brain iron accumulation, rare disease, mitochondrial dysfunction, oxidative stress, neuroinflammation, iron metabolism, lipid metabolism, autophagy, membrane remodelling

## Abstract

The syndromes of neurodegeneration with brain iron accumulation (NBIA) encompass a group of invalidating and progressive rare diseases that share the abnormal accumulation of iron in the basal ganglia. The onset of NBIA disorders ranges from infancy to adulthood. Main clinical signs are related to extrapyramidal features (dystonia, parkinsonism and choreoathetosis), and neuropsychiatric abnormalities. Ten NBIA forms are widely accepted to be caused by mutations in the genes *PANK2*, *PLA2G6*, *WDR45*, *C19ORF12*, *FA2H*, *ATP13A2*, *COASY*, *FTL1*, *CP*, and *DCAF17*. Nonetheless, many patients remain without a conclusive genetic diagnosis, which shows that there must be additional as yet undiscovered NBIA genes. In line with this, isolated cases of known monogenic disorders, and also, new genetic diseases, which present with abnormal brain iron phenotypes compatible with NBIA, have been described. Several pathways are involved in NBIA syndromes: iron and lipid metabolism, mitochondrial dynamics, and autophagy. However, many neurodegenerative conditions share features such as mitochondrial dysfunction and oxidative stress, given the bioenergetics requirements of neurons. This review aims to describe the existing link between the classical ten NBIA forms by examining their connection with mitochondrial impairment as well as oxidative stress and neuroinflammation.

## 1. Overview of the NBIA Syndromes

The NBIA (neurodegeneration with brain iron accumulation) syndromes are rare diseases with an estimated prevalence of 1–3/1,000,000, characterized by progressive hypo- and/or hyperkinetic movement disorders with abnormal deposits of iron in the brain, primarily in the basal ganglia. These complex multisystem disorders exhibit extrapyramidal dysfunction, mainly dystonia, parkinsonism, and choreoathetosis. Cognitive decline and psychiatric disturbances are also core features. The progression of the disease leads to the loss of ambulation normally within 10–15 years after onset. Secondary effects such as aspiration pneumonia, rather than the neurodegeneration inherent to the disease, can cause death in most of cases. However, lifespan is variable, depending on the NBIA form.

Ten genes are classically accepted as NBIA genes [1] (Table 1). The two major forms are PKAN (pantothenate kinase-associated neurodegeneration; 35–50%; *PANK2* gene) and PLAN (*PLA2G6*-associated neurodegeneration; ~20%; *PLA2G6* gene), followed by MPAN (mitochondrial membrane protein-associated neurodegeneration; 6–10%; *C19ORF12* gene) and BPAN (β-propeller-associated neurodegeneration; 1–2%; *WDR45* gene). FAHN (fatty acid hydroxylase-associated neurodegeneration; *FA2H* gene), NF (neuroferrinopathy; *FTL* gene), aceruloplasminemia (*CP* gene) and Woodhouse–Sakati syndrome (*DCAF17* gene) are rare types. Finally, two probands and five probands, respectively, are described for Kufor–Rakeb syndrome (*ATP13A2* gene) and CoPAN (COASY protein-associated neurodegeneration; *COASY* gene). Importantly, a relevant number of patients with NBIA have no genetic diagnosis, suggesting that other implicated genes remain to be discovered.

The abnormal accumulation of iron in the brain could be caused by a primary molecular defect or be the consequence of a secondary underlying metabolic defect. In the literature, we can find patients with clear-established NBIA clinical pictures as well as isolated patients with new genetic entities who may also present with NBIA (Table 2). Only the description of additional cases that allow a better genotype–phenotype correlation will make it possible to distinguish between NBIA entities and others, which in some unusual cases show NBIA, likely caused by a secondary effect.

The genetic heterogeneity and the corresponding encoded proteins emphasize that quite different disease mechanisms underlie NBIA conditions. Several pathways should be considered: iron and lipid metabolism, membrane remodelling, coenzyme A (CoA) synthesis, and autophagy. Notwithstanding, mitochondrial dysfunction is commonly implicated in neurodegeneration. Energy in the cell is mainly produced by oxidative phosphorylation within mitochondria, and this is indispensable for plenty of processes, including energy metabolism, calcium homeostasis, lipid biosynthesis, and apoptosis [19]. The bioenergetic requirements of neurons for cellular processes such as synaptic plasticity and neurotransmitter synthesis mean that they rely on the mitochondria [20]. The brain is especially sensitive to oxidative stress and damage, due to its high oxygen consumption (20% of body basal oxygen), low antioxidant defenses, and high content of metal ions [21]. Because of this, neurodegenerative disorders usually share common features such as the involvement of oxidative damage and mitochondrial dysfunction. In fact, most of the genes involved in neurodegenerative disorders such as Parkinson’s disease or amyotrophic lateral sclerosis (ALS) are related to mitochondria. All aggregated misfolded proteins (β-amyloid, tau, and α-synuclein) are known to inhibit mitochondrial function and induce oxidative stress [22]. Several NBIA genes are directly associated with mitochondria (*PANK2*, *COASY*, *PLA2G6*, and *C19ORF12*), showing the essential role of these organelles in triggering the pathological process.

Reactive oxygen species (ROS) production should be in balance with antioxidant processes in the cell; when it is not, oxidative stress occurs. Inflammation is a protective response against infection and injury that repairs and regenerates damaged tissue or cells and removes pathogens. However, ROS overproduction may lead to an exaggerated inflammatory response, the release of neurotoxic factors and ultimately, the loss of neuronal structure and function. Neuroinflammatory processes can be linked to multiple pathways, including the ones that lead to depression and dementia, both consequences of neurodegeneration. The underlying disease mechanism involves proinflammatory cytokines, and therefore, neuroinflammation is involved in the pathogenesis of functional and mental impairments [23]. Most NBIA entities may present with neuropsychiatric abnormalities and obsessive-compulsive behavior, which may be related to neuroinflammatory processes.

**Figure 1:**
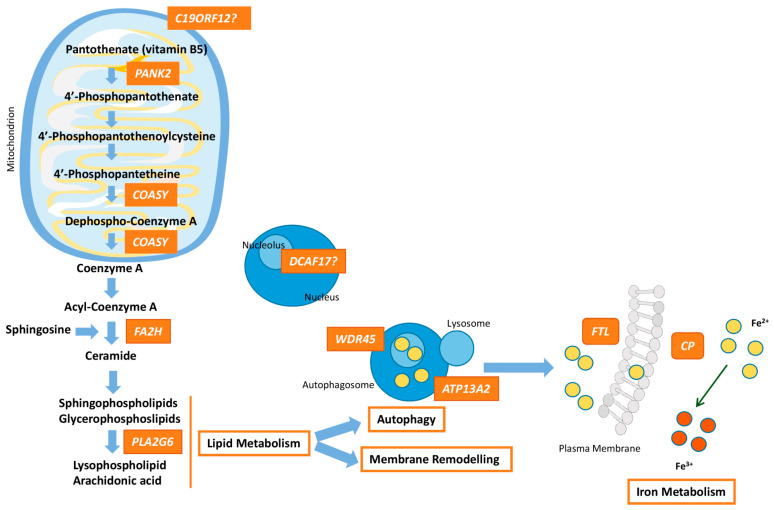
Pathway connecting lipid synthesis, lysosomes dysfunction and iron accumulation in NBIA syndromes. *PANK2, COASY, FA2H* and *PLA2G6* defects lead to harmed phospholipid membrane synthesis and impaired myelination or myelin maintenance. Dysfunctional membranes may lead to lysosomal and mitochondrial damage causing ROS production and iron uptake upregulation. *WDR45* and *ATP13A2* are involved in autophagosome formation/degradation. The decrease of WDR45 protein expression causes the accumulation of aberrant autophagic structures. Lysosomal dysfunction due to *ATP13A2* defect, which may originate from impaired phospholipid recycling, could ultimately cause degradation of substrates and damage of autophagosome clearance. Moreover, perturbation of lysosomes is important for iron homeostasis and may promote deposits of iron. Dysfunction of *FTL* prevents the recruitment of iron excess and malfunctioning of *CP* causes impairment of iron export from the cell, responsible for iron overload. The higher free iron-dependent oxidative damage requires that the cell increases the degradation of oxidized molecules, which can affect the cellular recycling systems, such as lysosomes, and finally, lead to cellular death. The role of *DCAF17* remains unclear, although its dysfunction leads to a neurodegenerative disorders, and hence, in some way, *DCAF17* may be related to mitochondrial dynamics.

In this review, we focus on the classical ten NBIA forms in which the resulting mutated proteins impair the mitochondrial function and induce oxidative stress, ultimately causing neuroinflammation (Figure 1).

## 2. NBIA Errors of Coenzyme a Biosynthesis

### 2.1. Pantothenate Kinase-Associated Neurodegeneration (PKAN)

Among NBIA disorders, the most common form is caused by biallelic mutations in the pantothenate kinase 2 (*PANK2*) gene (Table 1), which are responsible for PKAN, an autosomal recessive (AR) disorder [24,25,26,27,28]. Two main subtypes are associated with PKAN: classical (early onset, usually before 6 years of age) or atypical (first symptoms in early adulthood). The classical presentation is characterized by dystonic tremor with predominant oromandibular involvement, optic atrophy, pigmentary retinopathy, acanthocytosis, and spasticity. In the late-onset PKAN form, motor involvement tends to be less severe but cognitive decline and psychiatric alterations are predominant traits [29,30,31,32,33,34,35].

In PKAN patients, the characteristic “eye of tiger sign” is detected by T2-weighted magnetic resonance, which reflects the focal accumulation of iron in the globus pallidus (GP); adjacent structures and subcortical white matter are affected to a lesser degree [36,37,38]. The iron accumulation correlates with neural damage, often associated with neuroaxonal spheroids that represent degenerating neurons in GP [39,40]. In these abnormal structures, there is an accumulation of ubiquitin and other proteolytic processing markers, but Lewy bodies or α-synuclein (α-syn) are not present, moving away the PKAN neurodegeneration pathway from other well-known neurodegenerative diseases [41]. In addition, positive-ubiquitin axonal spheroids in pallidal neurons co-localize with apolipoprotein E (apoE), which was described as a marker for acutely ischemic hippocampal pyramidal and cortical neurons [42]. In addition to this, neuronal degeneration promotes a mild inflammatory response due to infiltrations of iron-containing macrophages, astrogliosis and microglial activation [41]. These findings suggest that chronic neuronal hypoxia and/or ischemia in the GP may play an important role in the pathomechanism of PKAN.

*PANK2* encodes for the mitochondrial isoform of pantothenate kinase that catalyzes the ATP-dependent phosphorylation of pantothenate ((R)-pantothenate into (R)-4′-phosphopantothenate using ATP), an essential regulatory step in CoA biosynthesis (Figure 1). The PANK2 protein, located in mitochondria, has two domains: one that includes two MLS (mitochondrial localization signals) in the NH_2_-terminal and the other large one that encodes for the catalytic core of the enzyme [40,43]. To date, more than 120 disease-causing mutations are known in *PANK2* (HGMD^®^ Professional 2020.1; accessed 8 October 2020). The most frequent type of PKAN mutation is the missense/nonsense, followed by splicing variants, small insertions or deletions and gross deletions. All of the known mutations are located in the catalytic domain, highlighting the relevance of the PANK2 enzymatic function in PKAN pathophysiology. Because PKAN is an AR-inherited entity, the expected explanation for the cause-effect of the *PANK2* mutations is total loss or partial deficiency in the enzyme activity [24,25]. In fact, a link between functionality of the mutated enzymatic domain and onset/severity of the clinical picture has been established [27,44]. However, other studies have demonstrated that many *PANK2* mutations do not disturb the protein function, suggesting alteration of dimerization dynamics or protein mislocation as putative disease mechanisms [45,46,47].

PANK2 acts as a sensor of mitochondrial CoA, and is tightly regulated by feedback inhibition by CoA and its thioesters [47,48]. The CoA fluctuation is released from β-oxidation of triglycerides. In a pathogenic scenario, this controlled process would be unbalanced, causing, among other alterations, deficient energy production, impaired biosynthesis and replacement of phospholipids in cell membranes, and an increase of oxidative stress [49,50]. Supporting this, elevated levels of mitochondrial dysfunction markers and reduced levels of different lipids (triglycerides, cholesterol metabolites and sphingomyelins) have been found in metabolic profiles of PKAN plasma samples [51]. There is a strong connection between alteration of CoA levels, ROS production and apoptosis. In fact, the PKAN’s pathomechanism is directly related to the overproduction of ROS and unbalanced mitochondrial redox, which may trigger a neuronal death cascade [52]. Particularly, in neuronal cells from induced pluripotent stem cells (IPSCs) of PKAN’s patients, lipid peroxidation and alteration of oxidative status (increased ROS production), mitochondrial impairment (including defects in mitochondrial respiration and electrophysiological properties) and premature cell death have been detected [53,54]. Little is known about why mutated *PANK2* induces iron accumulation in the brain, but a possible explanation would be the alteration of the mRNA expression of the iron exporter ferroportin (FPN) induced by *PANK2*-silencing found in HeLa cells [55]. Together with the hormone hepcidin, FPN is part of the hepcidin–FPN axis that is considered the key regulator of systemic iron homeostasis [56,57].

The most relevant insight into the biological mechanism connecting defects of *PANK2* and neurodegenerative processes has come from the advanced animal models of the disease developed in organisms such as flies, mice or zebrafish. In zebrafish, *pank2* has been observed to be expressed at high levels in the brain and particularly in neurons. Morpholino-mediated *pank2* down-regulation causes abnormal development of the central nervous system (CNS) and vascular structures. Additionally, a strong activation of the neuroinflammation processes has been developed with the loss of neural cells in fish’s telencephalon, the homologous area to human GP [58]. In *Drosophila melanogaster*, there are several transgenic flies available for *fumble* (*fbl*), the orthologue of the human *PANK2*. The dPANK/*fbl* hypomorphic model suffers from neurodegeneration in retinal tissues and in brain but without iron overload, lipid dyshomeostasis, mitochondrial dysfunction or increased protein oxidation [59]. Interestingly, in null *fbl* flies, the human *PANK2* and other PANK isoforms (*PANK3* and *PANK4*) are able to restore the WT (wild-type) phenotype [60].

*Pank2* depletion in knockout (KO) mice leads to growth retardation, azoospermia and retinal degeneration. Brain iron deposits, movement disorders or neurodegenerative signs are not displayed, unless they are subjected to a ketogenic diet [61,62]. Neurons derived from adult KO mice and the neonatal hippocampus show an altered mitochondrial membrane potential and deficient mitochondrial respiration, both of them accompanied by an increase in ROS generation [63]. Another study in KO mice showed mitochondrial dysfunction, defects in CoA metabolism, and an increase of iron levels specifically in cells isolated from GP [64]. Using Hopantenate (HoPan), as a competitive inhibitor of the murine PANKs, the CoA levels were drastically reduced. In this model, the liver was the most affected organ while the brain showed normal histology compared to the control animals [65]. Another alternative is the deprivation of pantothenic acid (PA), the substrate for PANK2, which consists of a lack of this compound in the mice diet. In this case, animals exhibit a phenotype characterized by azoospermia, growth retardation, skin and eye lesions, and movement disorders. Interestingly, the addition of PA to the diet can rescue the WT phenotype, except for the growth problem [66]. More recently, a complete elimination of CoA in the brain has been achieved with a double KO mouse: a systemic deletion of *Pank1* combined with a conditional deletion of *Pank2* in neurons (both involved in the regulation of CoA levels). This double KO manifests a clear motor dysfunction, an alteration of brain hemoglobin synthesis and a dysregulation of genes involved in hypoxic cellular pathways or ischemic injury [67]. Despite all of the promising advances regarding the PKAN molecular bases, an effective pharmacological treatment is not available yet. Research is mainly aimed at decreasing iron levels in the GP and in bypassing the defective PANK2 enzyme. Deferiprone (an iron chelator), which is able to cross the blood–brain barrier, decreases the accumulated iron in pallidal neurons [68,69,70,71]. Recently, a review on clinical trials using deferiprone has reflected that although accumulated iron decreased in the GP, motor symptoms, such as dystonia, do not seem to improve: the effect of deferiprone is practically non-existent in patients with the classical phenotype, whereas patients with the atypical form present with a slower progression [72]. Regarding PANK2 activity, direct supplementation of pantothenate is effective in high doses in KO mice; however, a CoA deficiency is produced in many tissues by inhibition of the rest of PANKs [73], and as expected, pantothenate has no effect on fibroblasts derived from patients with truncated PANK2 [74]. Different compounds have been tested in order to increase the CoA levels without having to go through the classic PANK2 route. In dPANK/*fbl* flies, the WT phenotype was rescued for locomotor disabilities, mitochondrial impairment and brain degeneration, by using pantethine as an alternative CoA precursor [75]. Pantethine also improved histological and motor symptoms and reversed the mitochondrial damage in derived neurons from a *Pank2* KO murine model fed with a ketogenic diet [61]. In addition to these two therapeutic methods and in agreement with the guideline of clinical approaches for PKAN patients, medication management is primarily symptomatic: drugs (baclofen, clonazepam or hexyphenidyl) and other specific approaches such as botulinum toxin or deep brain stimulation are used to improve focal dystonia [76].

### 2.2. COASY Protein-Associated Neurodegeneration (CoPAN)

CoA synthase, a bifunctional enzyme that catalyzes the last two steps in CoA biosynthesis, is encoded by the *COASY* gene (Figure 1). The protein contains a mitochondrial localization signal, a regulatory region and two domains for its catalytic kinase activities, 40PP adenyltransferase (PPAT) and dephospho-CoA kinase (DPCK), and it is predominantly located at the mitochondrial matrix. Three different splice variants were described in humans: COASY-α showing ubiquitous expression, COASY-β being brain-specific, and COASY-γ probably coding for the COOH-terminal DPCK domain.

Mutations in this gene lead to COASY protein-associated neurodegeneration (CoPAN), an ultra-rare NBIA form inherited in an AR manner (Table 1). Hitherto, four CoPAN families are known [77,78,79] and three missense variants have been described (HGMD^®^ Professional 2020.1; accessed 10 September 2020). Two independent Italian cases that shared the p.R499C mutation were reported: in one family, the proband was homozygous for the mutation, while in the other family, the affected individual was compound heterozygous for p.R499C and p.Q59*. Patients’ fibroblasts show a reduction in the amount of CoA synthase and also CoA, compared to controls, suggesting that the mutation that affects the stability of the protein and, therefore, its role in the CoA biosynthesis pathway are impaired. Nevertheless, why only the brain seems to be affected while this enzyme is expressed in all tissues requires further investigation [77]., further additional patients have been reported: an Italian patient from a consanguineous family carrying the p.R499C mutation in homozygosis [78], and two Turkish siblings who were compound heterozygous for p.R499C and p.A214V [79]. All these patients show typical NBIA features: onset in the first decade of life with mild cognitive impairment and gait difficulties, progressing to a more severe phenotype including dystonia, parkinsonism, dysarthria, spasticity and axonal neuropathy. Obsessive-compulsive disorder is also a common feature in CoPAN patients.

Although two genes, *CAB4* and *CAB5*, are responsible for the last steps of CoA biosynthesis in yeast, the human *COASY* can complement the deletion of CAB5. The evaluation of the consequences of the p.R499C mutation using a yeast model has revealed a reduction in the respiration rate and a decrease in the steady state levels of the respiratory chain subunits. Moreover, the mutant strains show an increased sensitivity to H_2_O_2,_ pointing out an iron excess and oxidative stress. Levels of succinate dehydrogenase, a mitochondrial marker linked to iron metabolism, is decreased in the mutants. Lipid content is lower in the mutants compared to the controls, suggesting a perturbation of the lipid homeostasis probably due to a reduction in the CoA levels [80].

*Drosophila melanogaster* mutants with defects in the first (dPANK/fbl), second (dPPCS) and final (dPPAT-DPCK) enzymes of the CoA synthesis pathway show alterations in lipid homeostasis, shorter life span, locomotor dysfunction, an increase in ROS sensitivity and, surprisingly, an impairment in DNA integrity, which could be linked to abnormalities during the development of the CNS [81].

In zebrafish, *coasy* is widely expressed from the earliest stages of development and it shows high similarity to its human ortholog. A model developed with a morpholino-mediated approach has revealed that a reduction in the CoA content, an increase in the mortality, and a phenotype similar to that of dorsalized mutants is a consequence of the complete abolition of *coasy* expression. Milder phenotypes from lower doses of morpholino lead to neurodevelopmental abnormalities, vascular arborisation and a decrease in the expression of bone morphogenetic protein (Bmp) receptors alongside higher cell death [82]. The observed phenotype can be rescued by the overexpression of the WT human gene and the supplementation of the fish water with CoA, demonstrating the specific role of appropriate CoA levels and pointing to the Bmp pathway as a possible molecular linker in the disease.

The CoA biosynthesis pathway and lipid metabolism are anomalous in CoPAN patients. CoA functions as an acyl transporter, allowing its transfer to the target molecules. For instance, holo-mtACP is the active form of the mitochondrial acyl carrier protein and stands as a key component in mitochondrial fatty acid synthesis. The impaired biosynthesis of CoA results in lower holo-mtACP levels, together with a reduction in pyruvate dehydrogenase activity (PDH), in *Drosophila melanogaster* and mammalian cells [83]. The PDH activity may be rescued by stimulation with dichloroacetate, a compound that suppresses a PHD inhibitor, suggesting that clinical signs related to defects in the CoA-mtACP-PDH pathway can be eased. In line with this, the use of deuterated polyunsaturated fatty acids is proposed to reduce lipid peroxidation according to the beneficial findings in *Drosophila* [84]. Further investigation is needed to provide insights into the connection between CoA biosynthesis and neurodegeneration in order to achieve a cure for CoPAN patients.

## 3. NBIA Types Related to Lipid Metabolism and Membrane Remodeling

### 3.1. PLA2G6-Associated Neurodegeneration (PLAN)

AR mutations in the phospholipase A2 group (*PLA2G6*) are causative of the PLAN phenotypic spectrum (Table 1), including classic infantile neuronal dystrophy (INAD), atypical neuronal dystrophy (NAD) with childhood-onset, and an adult onset dystonia-parkinsonism form named PARK14 [85,86]. INAD has an early-onset (between 6 months and 2 years old). This NBIA type is characterized by progressive motor deterioration and may lead to spastic or hypotonic tetraparesis with truncal hypotonia, cerebellar ataxia, early optic atrophy, seizures in later stages of the disease, dystonia, and cognitive impairment [87]. The most typical sign of INAD is a fast progression of cerebellar atrophy in early stages. At later stages of the disease progression, most INAD patients usually show brain iron accumulation in the GP and the substantia nigra [88,89]. About 200 distinct mutations in *PLA2G6* of all types (missense, deletions, frameshift, nonsense, splice site), including multiexon deletion and duplication [90,91,92], have been published (HGMD^®^ Professional 2020.1; accessed 15 September 2020). Because PLAN is caused by biallelic mutations, the disease mechanism is expected to be caused by loss of function.

In patients with later disease onset, the phenotype may be atypical NAD or dystonia-parkinsonism. They usually show pyramidal signs, eye movement abnormalities, cognitive decline and psychiatric features. Dystonia-parkinsonism, characterized by the presence of tremor, rigidity, and severe bradykinesia, is associated with Lewy bodies and NAD. Interestingly, these patients do not usually present with cerebellar atrophy [93,94]. The pathological hallmark of PLAN is the presence of spheroids throughout the nervous system located in peripheral axons and nerve terminals [95], which are composed by accumulation of membranes with tubulovesicular structures (TVS) that are usually positive for α-syn and ubiquitin [96,97,98].

*PLA2G6* encodes several isoforms of VIA calcium-independent phospholipase A2 (iPLA2β), which hydrolyzes the sn-2 ester bond in membrane phospholipids, releasing free fatty acids and 2-lysophospholipids [99,100] (Figure 1). iPLA2β is involved in transduction and maintenance of phospholipid homeostasis, releasing docohexaenoic acid (DHA) and arachidonic acid [101]. In addition, roles related to inflammation and immune responses, chemotaxis, vascular relaxation, secretion and apoptosis have been described for iPLA2β [102,103].

iPLA2β is expressed throughout the mammalian brain with a high expression in neurons, in dendrites and axon terminals, suggesting a role in neuronal signaling [104,105]. iPLA2β is usually distributed in the cytosol and in mitochondria with variable subcellular localization to the nucleus, Golgi and endoplasmic reticulum (ER) in mammalian cells [104,106,107,108,109]. Dysfunction of iPLA2β alters dynamics of cellular membranes, retrograde trafficking, endosome recycling and assembly, and maintenance of TVS of the Golgi complex [110,111].

The details of neuronal dysfunction and neurodegeneration mechanisms associated with mutations in *PLA2G6* remain unclear. Several iPLA2β KO mice models showed disturbances in brain phospholipid metabolism and in Ca^2+^ signaling in astrocytes associated with mitochondrial impairment [112,113]. The addition of disturbance phospholipids ameliorated the disturbances and partially restored the mitochondrial function [114,115,116].

iPLA2β deficiency leads to insufficient remodeling and degeneration of mitochondrial and presynaptic membranes. There is evidence of mitochondrial injury in iPLA2β KO mice models. Furthermore, patients’ fibroblasts and iPLA2 KO *Drosophila* models show increased mitochondrial lipid peroxidation. PLAN models show, at least at the late stage of disease, presynaptic membrane and mitochondrial degeneration, with progressive accumulation of TVS and spheroids containing ubiquitinated proteins through the entire brain, particularly in distal axons, as seen in patients [97,117].

The inner mitochondrial membrane contains a high proportion of cardiolipin, which is susceptible to attack from ROS due to the high content of fatty acids. This would bring about a downstream pathogenic cascade of events involving release of cytochrome c and apoptosis [118]. An iPLA2 *Drosophila* model shows altered mitochondrial inner membrane protein expression levels and morphology at 15 weeks of age, but there is no evidence of axonal degeneration. The progression is age-dependent, where the mitochondrial membranes are gradually degenerated, so double membrane ruptures and damaged axons are probably associated with the release of cytochrome c and ROS outside mitochondria. Furthermore, the presence of the mitochondrial HNE (4-hydroxy-2-nonenal), a reactive lipid peroxidation product, indicates the presence of an essential activity in mitochondria [96,118]. Therefore, insufficient remodeling and degeneration of mitochondrial inner membranes and presynaptic membranes may appear to be the cause of disease.

Several animal models have demonstrated that expression of iPLA2β prevents the loss of mitochondrial membrane potential and attenuates the cytochrome c release, the decrease of ROS and mitochondrial lipid peroxidation in response to stress-induced apoptosis, protecting mitochondria. The apoptotic cascade, ROS and lipid peroxides are enough to originate a neuroinflammation response, which may potentiate PLAN pathogenesis [106,119,120,121].

In an iPLA2 KO mouse, cerebellar atrophy with significant loss of Purkinje cells has been observed at the age of 13 months. Before degeneration, mice showed a reactive astrogliosis and microglial activation with a pronounced cytokine up-regulation [122]. Hence, neuroinflammation may lead to neuronal death provoked by mitochondria damage and may strongly contribute to the pathological development of cerebellar atrophy in PLAN disease. In addition, disruption of brain DHA levels in aged KO mice cause microglial and astrocytic activation, motor disturbances and cerebellar neural loss by 15–20 months, leading to increased neuroinflammation [123]. Oxidative stress and neuroinflammation could potentiate each other to promote the progression of PLAN disease.

Treatment is mainly symptomatic in PLAN patients. There is some basis to recommend DHA supplements, especially given their low toxicity [124]. A clinical trial using deutered polyunsaturated fatty acids is underway to assess efficacy; they may be able to partially rescue the locomotor problems and restore mitochondrial potential, by inhibiting lipid peroxidation [125,126]. In addition, early anti-inflammatory therapy may help to slow down the progression of cerebellar atrophy in PLAN patients.

### 3.2. Mitochondrial Membrane Protein-Associated Neurodegeneration (MPAN)

Mutations in the *C19ORF12* (chromosome 19 open reading frame 12) gene cause MPAN (Table 1), which is characterized by cognitive decline progressing to dementia, speech and gait disturbances, parkinsonism, optic atrophy and motor axonal neuropathy [127]. The first *C19ORF12* mutation discovered was a homozygous 11 bp deletion leading to a truncated protein (c.204_214del11, p.Gly69Argfs*10)) in 13 families from Eastern Europe [128]. Nowadays, about 50 *C19ORF12* variants have been found to relate to the MPAN phenotype, including missense/nonsense, indels and splicing mutations (HGMD^®^ Professional 2020.1; accessed 7 October 2020). Although MPAN is considered an AR condition, a single *C19ORF12* mutation can lead to the same clinical phenotype as biallelic variants [129]. The mutations with AR inheritance are degraded by the NMD (nonsense-mediated decay) system, generating haploinsufficiency. In contrast, the mutations with autosomal dominant (AD) inheritance may be located in the final part of *C19ORF12* mRNA and may escape from NMD. Therefore, the resulting protein would have a dominant negative effect on the WT that may explain the duality in MPAN inheritance [129]. Interestingly, alternative clinical phenotypes to MPAN have been reported for distinct *C19ORF12* variants: the c.187G>C (p.A63P) mutation causes an AR form of spastic paraplegia with amyotrophy (SPG43) [130,131], the c.197-199del3 variant leads to MPAN phenotype aggravated with traits of juvenile amyotrophic lateral sclerosis (ALS) [132], and a clinical picture of Karak pallido-pyramidal syndrome has been described for the c.157G>A (p.G53R) mutation [133].

*C19ORF12* synthesizes a small transmembrane protein (17 kDa), whose function remains unclear (Figure 1). It is widely expressed in the brain and adipocytes and localized in the lumen and MAMs (mitochondria associated membranes) of mitochondria, and in the ER [128,131,134]. In HeLa cells exposed to H_2_O_2_, the WT protein forms cytosolic aggregates, while C19ORF12 p.G58S and p.Q96P are retained in the mitochondria and ER, being insensitive to oxidative stress [134]. Similarly, in vitro analysis of fibroblasts exposed to H_2_O_2_ shows an increase in mitochondrial Ca^2+^ accompanied by a greater activation of the apoptotic rate in patients’ fibroblasts compared to control cells [134]. These two results demonstrate that ROS deregulation plays vital roles in the MPAN pathogenic mechanism. Transgenic *Drosophila* models of MPAN manifested in a shorter lifespan and locomotor impairment, but the most remarkable trait was the accumulation of degenerative vacuoles in the brain [135]. In patients, in addition to the classical iron accumulation in SN and GP, post mortem brain examination revealed neuroinflammation marks such as α-synuclein-positive Lewy bodies, axonal spheroids and neuronal inclusions containing hyperphosphorylated tau protein [128,136,137].

Despite the existence of a link between oxidative stress, neuroinflammation and MPAN pathophysiology, it is unclear how mutations in *C19ORF12* orchestrate the aforementioned phenotype. There are different hypotheses to explain this fact. Between them, C19ORF12 would act as a regulatory protein for human MgtE transporters and the alteration of magnesium has been related to the progression of many neurodegenerative disorders [134,138]. More recently, a correlation among ferroptosis and MPAN has been established by the fact that both pathological situations share lipid peroxidation and mitochondria perturbation as major pathways of cell death [139].

### 3.3. Fatty Acid Hydroxylase-Associated Neurodegeneration (FAHN)

Mutations in *FA2H* (fatty acid 2-hydroxylase) cause AR fatty acid hydroxylase-associated neurodegeneration (FAHN) (Table 1). Clinically, FAHN is characterized by ataxia, dystonia, spasticity, ocular abnormalities, cerebellar atrophy, and iron deposition, predominantly in the GP. Cognitive impairment and seizures may be features of the disease [140]. White matter abnormalities, a thinner corpus callosum and supratentorial atrophy seem to be hallmarks shared by the vast majority of the patients [141]. Up to 65 mutations (missense/nonsense, frameshift, splicing and indels) in *FA2H* have been described (HGMD^®^ Professional 2020.1; accessed 7 October 2020). Additionally, *FA2H* is also involved in familial leukodystrophy and hereditary spastic paraplegia (SPG35).

*FA2H* encodes a NADPH-dependent mono-oxygenase that colocalizes with the ER membrane. *FA2H* is crucial during the early stages of brain development. Due to its 2-hydroxylase activity, *FA2H* produces 2-hydroxylated ceramides and therefore, participates in myelin formation [142] (Figure 1). A mice model deficient in *FA2H* lacks 2-hydroxilated sphingolipids in brain and peripheral nerves, accompanied by myelin sheath degeneration and axonal impairment in both the spinal cord and sciatic nerves [143]. Other mouse mutants, with either a deficiency of this gene in all cells or only in oligodendrocytes and Schwann cells, show demyelination, structural alterations and degeneration of the axons [144]. Nonetheless, why the deficiency of *FA2H* causes abnormal brain iron accumulation is poorly understood.

Mutations affecting the cytochrome b5-like heme binding domain and the fatty acid hydroxylase domain result in a reduction of the enzyme’s activity, as well as the possible disruption of the interaction of these domains with iron-containing ligands [145]. The reduction of the enzymatic activity or its complete depletion, caused by the different mutations located throughout the protein, could explain the variability in the severity of the phenotypes. Further investigation to elucidate the underlying disease mechanism is required to develop an adequate therapy, as nowadays treatments are restricted to ameliorating the symptomatology [139].

## 4. Autophagosome/Lysosome Regulation

### 4.1. β-propeller-Associated Neurodegeneration (BPAN)

β-propeller protein-associated neurodegeneration (BPAN) disorder is caused by mutations in the X chromosome gene *WDR45*, which is transmitted in an AD manner (Table 1). Originally, the description of BPAN patients has been referred to as static encephalopathy of childhood with neurodegeneration in adulthood (SENDA), but BPAN is currently established as a NBIA form [146,147]. To date, all affected individuals are sporadic cases with no family history (de novo mutations). Clinical features do not always follow the typical pattern for an X-linked disorder. Apparently, affected men, who are carriers of hemizygous *WDR45* mutations, are predicted to harbor post-zygotic mutations, suggesting that male patients could be somatic mosaicisms [146]. Affected women may carry either germline or somatic mutations, showing different phenotypic manifestations, probably associated with skewing of X chromosome inactivation [148]. So far, about 112 disease-causing mutations are known in *WDR45* (HGMD^®^ Professional 2020.1; accessed 13 October 2020).

Clinically, BPAN is well characterized as a two-stage disease progression. The first stage comprises a global developmental delay in childhood with intellectual disability. Common early comorbidities comprise seizures, spasticity, and epilepsy. The second stage affects all patients in early adulthood, and manifests with progressive dystonia, dementia and parkinsonism characterized by bradykinesia and rigidity without tremor [146,147,148].

Brain magnetic resonance imaging (MRI) shows iron accumulation in the substantia nigra and globus pallidus in the early phase. A T1-weighted hyperintense “halo” signal with a central band of hypointensity in the substantia nigra seems to be a specific finding in BPAN. Cerebral atrophy is also reported in most patients. Additional symptoms are sleep disturbance, ocular features and Rett-like hand stereotypies [148].

Defects on *WDR45* produce loss of protein coding function and impairment of autophagy leading to BPAN neurodegeneration (Figure 1), although the detailed mechanism needs to be clarified. The WDR45 protein is a member of the WD40 repeat protein family with a β-propeller platform structure. WD40 proteins play a role in coordinating protein–protein interactions in order to perform a variety of functions, such as signal transduction, autophagy or transcriptional regulation. In particular, the WDR45 protein, by binding to phosphatidylinositol-3-phosphate (PtdIns3P), regulates autophagosome formation [149,150,151]. In this way, defective autophagic flux associated with *WDR45* mutations have been described in different cellular and animal studies [147,152,153,154,155]. KO mice for *Wdr45* show an impaired autophagic flux with accumulation of SQSTM1- and ubiquitin-positive aggregates in neurons and swollen axons. Furthermore, KO male animals displayed learning and memory defects and axonal swelling, although without iron deposition in the basal ganglia [151]. In line with this, accumulated ER proteins have been observed as a result of WDR45 deficiency, together with increased ER stress and impaired ER quality control. Suppression of ER stress or activation of autophagy prevents cell death. In addition, loss of neurons is present in aged mice and increased apoptosis indicates neurodegeneration. Iron levels present no changes at six month of age, but significant differences have been detected at an older age, indicating neurodegeneration [152].

In BPAN patients’ fibroblasts, a positive regulation of the iron transporter IRE/DMT1 (iron responsive element–divalent metal transporter 1) and a negative regulation of the transferrin receptor have been found, like in other NBIA disorders [153]. In addition, an increase in intracellular Fe^2+^ after starvation have been observed, which may explain the accumulation of iron in the basal ganglia due to defects in autophagy and recycling, and alterations in lysosomal degradation [153]. IPSC-derived midbrain neurons together with fibroblasts from a BPAN woman showed augmented levels of intracellular iron and reduced levels of L-ferritin, H-ferritin and mitochondrial ferritin. Oxidative stress accompanies these effects by mitochondrial abnormalities, as well as autophagy defects, and reduces lysosomal function. Phenotype is restored when the WDR45 levels are increased [154].

### 4.2. Kufor–Rakeb Syndrome

Kufor–Rakeb disease (KRD) is a very rare early-onset atypical parkinsonism caused by mutations in the *ATP13A2* gene with AR inheritance (Table 1). Symptoms appear before 20 years of age and are characterized by motor symptoms (pyramidal degeneration) and non-motor symptoms (dementia, learning difficulties and hallucinations) [156,157]. About 50 mutations associated with KRD, including additional phenotypes such as neuronal ceroid-lipofuscinosis and hereditary mutations, have been reported in *ATP13A2* (HGMD^®^ Professional 2020.1; accessed 16 September 2020) [158,159]. Most patients with AR parkinsonism do not accumulate iron in the brain despite being clinically symptomatic. Nevertheless, some cases with homozygous mutations in *ATP13A2* and evidence of iron deposition in the basal ganglia have been reported, which makes it possible to include KRD in the NBIA group of disorders [160,161].

*ATP13A2* encodes for a lysosomal 5P-type ATPase, and is mostly localized in endosomes, lysosomes, and partially, in autophagosomes (Figure 1). Several functions have been attributed to ATP13A2 including homeostasis of manganese, zinc and iron, allowing active transportation across endosomal and lysosomal membranes, mitochondrial bioenergetics and the autophagy-lysosomal pathway [157,162,163,164]. Defects in *ATP13A2* may impair the endo-lysosomal and autophagy flux, resulting in the accumulation of insoluble proteins and damaged mitochondria, leading to apoptosis and neuroinflammation. Cellular models of patients with KRD have demonstrated increased mitochondrial fragmentation, oxidative stress, high ROS levels, DNA damage and ATP depletion [165,166].

Loss of ATP13A2 function is also associated with α-syn accumulation [166,167]. IPSC-derived dopaminergic neurons from KRD exhibit decreased secretion of α-syn from the axon and the cell body, as well as a disruption of the lysosomal Ca^2+^ homeostasis. Enhancing lysosomal exocytosis by overexpression of *ATP13A2* helps to control the α-syn levels [166,167].

The therapy for KRD patients is only symptomatic and similar to the treatment of Parkinson’s disease, mostly for motor symptoms. This therapy consists of the combination of levodopa and carbidopa, together with close evaluation of parkinsonism. Dopamine agonists or anticholinergics can be prescribed in some cases [168].

## 5. NBIA Forms Caused by Mutations in Iron-Related Genes

### 5.1. Aceruloplasminemia

Aceruloplasminemia is a rare AR disorder caused by mutations in the *CP* gene (Table 1), which encodes for ceruloplasmin (Cp), a multicopper ferroxidase functioning as an iron exporter from cells [169]. Intracellular Fe^2+^ is transported by ferroportin to transferrin via the ferroxidase activity of ceruloplasmin (Fe^2+^→Fe^3+^) (Figure 1). Close to 70 mutations (missense, frameshift, splicing, nonsense) have been described in *CP* (HGMD^®^ Professional 2020.1; accessed 16 September 2020). Homozygous mutations are identified in the vast majority of patients, although compound heterozygosity may be also detected [170]. Even though inheritance is AR, heterozygous carriers may present with a milder clinical picture [171].

Cp function is essential in astrocytes as it is the only ferroxidase in this cell type. Its absence causes remarkable morphological abnormalities, oxidative stress due to iron accumulation, and lipid peroxidation. These features are also present in other brain tissues, being severe in the basal ganglia, thalamus and cerebellum of the patients, which supports toxicity of iron excess [172].

The *CP*-associated phenotype is characterized by dementia, ataxia, chorea and parkinsonism in adulthood [173]. Microcytic anemia, liver disease and retinopathy are also common clinical features. Prior to the neurological symptoms, endocrine dysfunction may be present in some patients in the form of diabetes mellitus, abnormal glucose tolerance or abnormal MRI findings of the liver and the brain [174].

Transferrin-independent iron uptake studies have been performed in iron-loaded neuronal and glial cells, demonstrating the generation of ROS and cell death [175]. A *Cp* KO murine model has been described with hypoferremia and mild microcytic anemia accompanied with an excess of iron in the liver, while no overload has been detected in other tissues (i.e., brain) [176]. Iron deposition in KO mice reveals a similar pattern to that found in patients, with significantly increased iron accumulation in the brainstem, cerebellum, and retina. Lipid peroxidation is not a feature of the *Cp* KO mice’s cerebellum, although these cerebellar cells are sensitive to iron-mediated oxidative stress in vitro. Gradual iron accumulation over the long-term leads to progressive levels of cell damage and lipid peroxidation, so these products may be difficult to detect in the mice model in vivo. Reduction in cell culture viability may be rescued by desferrioxamine, a cell-permeable iron chelator, indicating that toxicity implies the production of toxic hydroxyl radicals caused by high iron levels. No accumulation of iron has been found in muscle or kidney, revealing that the phenomenon is tissue-specific [177].

Iron-chelating therapy reduces both hepatic and pancreatic iron overload, but this is not effective on brain iron overload. Although an early initiation of the treatment is associated with slower progression of the disease, other alterations such as anemia could occur in long-term treatments [170]. *CP* patients are treated with iron chelators (deferoxamine, deferiprone) in order to decrease serum ferritin concentration and brain and liver iron stores, although it is important to notice that deferoxamine is not able to cross the blood-brain barrier [174]. Regarding antibiotics, tetracyclines are able to cross the blood-brain barrier and have iron-chelating features [178]; minocycline has been reported as therapy to block the progression of neurologic signs [179]. Another approach could be the administration of oral zinc sulfate to prevent oxidative damage. This therapy leads to the induction of metallothionein synthesis, strengthening zinc’s antioxidant function. In consequence, oxidative stress related to iron overload may be severely reduced. Zinc, due to its tolerability without side effects and its capability to stop iron absorption, stands as a valuable option [180].

### 5.2. Neuropherritinopathy (NF)

Mutations in the ferritin light chain *(FTL)* gene cause neuroferritinopathy, an AD inherited disease with adulthood onset (Table 1). So far, ten nucleotide duplications in exon 4 (HGMD^®^ Professional 2020.1; accessed 10 September 2020), involving COOH-terminal residues of the protein, are associated with NF, and they may account for dystonia, dysarthria, cerebellar signs, parkinsonism, chorea and psychiatric features [181].

Ferritin removes ferrous iron from labile iron pools in cells in order to prevent cellular damage, ROS formation, lipid peroxidation, protein aggregation and iron overload, common features of NF (Figure 1). Iron is stored in the form of ferric iron oxide inside ferritin, which is responsible for its release when it is required by cellular pathways.

Native human ferritin is a heteropolymer composed of two subunits: FTH1 and FTL. Each cell/organ presents different ratios of these subunits depending on their storage rate and optimization. COOH-terminus modifications seem to result in an alteration of the heteropolymeric structure that prevents iron from binding, and promotes the generation of ferritin aggregates and the formation of inclusion bodies [182]. As this is a consequence of long-term stress conditions, development of the disease is gradual in the patients.

In vitro studies with patients’ fibroblasts have showed increased production of ROS and ferritin polypeptides, iron overload and reduction in transferrin receptor levels and iron regulatory protein-iron responsive element binding activities [183]. These results are similar to those obtained with a transgenic mice model; while the iron levels are slightly increased in the mutant mice in comparison with controls, the overproduction and aggregation of ferritin as well as the evidence of oxidative damage are significant. In the same way, brain extracts from mutant mice revealed higher levels of lipid peroxidation, protein carbonyls and radical production [184].

Thus far, there is no treatment for NF. Neither patients treated with iron chelators, deferoxamine or deferiprone, nor monthly venesections show any improvement, although iron depletion is substantial [181]. The use of deferiprone in treating in vitro primary embryonic fibroblasts from a NF mice model resulted in a reduction of iron overload and of the cellular oxidative damage. Nonetheless, in vivo, this treatment affected iron homeostasis and ferritin deposition in mice without ameliorating the pathology, accordingly to the previous data from patients treated with iron chelators [185].

## 6. Another NBIA Subtype: Woodhouse-Sakati Syndrome

Woodhouse-Sakati syndrome is a rare AR disorder caused by mutations in the *DCAF17* gene (Table 1). To date, a total of 17 variants related to this syndrome have been described, including five missense mutations, six splicing mutations, and 12 small insertions or deletions (HGMD^®^ Professional 2020.1; accessed 8 October 2020). The complex clinical picture is characterized by the effects on SNC, but also on the SNP and the neuroendocrine system. Some of the Woodhouse-Sakati traits are hypogonadism, diabetes mellitus, mental retardation, deafness, alopecia, polyneuropathy and extrapyramidal impairment [186,187,188,189,190,191,192,193,194].

This gene encodes a nucleolar protein (Figure 1) expressed in many tissues, including the brain, liver, skin and gonads [186]. The function of Dcaf17 is still unknown, but it is related to the *Dcaf* gene family and is involved in apoptosis, DNA methylation and cell cycle regulation [195]. Fibroblasts of patients showed sensitivity to transcriptional blockade induced by actinomycin D, an inhibitor of nucleolar RNA polymerase I [186]. More recently, a crucial role in mammalian gonadal development and male reproduction has been described for Dcaf17 using a mouse model [194].

## 7. Conclusions

NBIA syndromes share the distinctive feature of iron overload in the brain and present a wide genetic heterogeneity with genes related to disparate pathways. How these different genes can cause the abnormal deposits of iron in the brain is a matter of investigation. However, as usually occurs in many other neurodegenerative disorders, mitochondrial dysfunction may play a vital role in the underlying pathomechanism because of the high energy demand within the brain. NBIA genes directly related to mitochondrial functions are *PANK2*, *COASY* and probably, *C19ORF12*, which ultimately together with *PLA2G6* and *FA2H* are involved in phospholipid membrane synthesis (Figure 1). *WDR45* is crucial in the formation/degradation of autophagosomes as well as *ATP13A2*, a lysosomal protein. Lysosomal activity is essential for iron homeostasis in which *FTL* and *CP* takes part. As a whole, the disease process in at least seven NBIA forms involves mitochondrial dysfunction, which produces oxidative stress and leads to neuroinflammation. Nonetheless, the list of NBIA genes is growing and the connection between all the players involved in NBIA disorders is unclear. Further studies are necessary with the aim of characterizing targets for therapeutic interventions.

## Figures and Tables

**Table 1:** The ten classical NBIA forms.

Gene (OMIM*)	NBIA form (OMIM#)—Inheritance Frequency	Clinical and MRI Findings	Protein	Function
*PANK2* (606157)	Pantothenate kinase-associated neurodegeneration (PKAN) (234200)—AR 35–50%	Onset is in early childhood, although atypical forms may occur at adolescence or early adulthood. Dystonia (oromandibular dystonia is prominent), parkinsonism, spasticity, pigmentary retinal degeneration, acanthocytosis, cognitive decline, neuropsychiatric disturbances. Hypointensity with central hyperintensity of the GP (“eye of the tiger”).	Pantothenate kinase 2	PANK2 takes part in the first step of the CoA synthesis: PANK2 catalyzes the ATP-dependent phosphorylation of pantothenate to 4-phosphopantethenate.
*PLA2G6* (603604)	PLA2G6-associated neurodegeneration (PLAN) (610217)—AR ~20%	Classical forms begin at infancy with progressive motor and mental retardation, marked truncal hypotonia, optic atrophy, pyramidal signs, and seizures. Atypical forms have an onset at adolescence or early adulthood, and include dystonia-parkinsonism, cerebellar ataxia, eye movement abnormalities, cognitive decline, and psychiatric disturbances. Hypointensity of the GP and in SN (in <50% of patients).	Phospholipase A2, group VI	PLA2G6 hydrolyses glycerophospholipids at the sn-2 position of acyl chains to produce lysophospholipids and free fatty acids, which participates in essential functions such as membrane remodelling, fatty acid oxidation, cell signaling, apoptosis, etc.
*C19ORF12* (614297)	Mitochondrial membrane protein-associated neurodegeneration (MPAN) (614298)—AR 6–10%	Childhood to early adulthood-onset with prominent neuropathy. Patients present with spastic para-or tetraparesis with muscle atrophy. Parkinsonism and dystonia are frequent. Optic atrophy, dementia, and psychiatric symptoms may happen. Hypointensity of the GP and SN plus hyperintensity in the GP.	Chromosome 19 open reading frame 12	Unknown function. Mitochondrial protein linked to lipid homeostasis because its expression follows fatty acid metabolism.
*WDR45* (300526)	β-propeller-associated neurodegeneration (BPAN) (300894)—XD 1–2%	Childhood type shows developmental delay and intellectual disability, mimicking an atypical Rett syndrome. In adulthood, patients present with dystonia-parkinsonism and dementia, together with seizures, and ataxia. Hypointensity of the GP and SN with central hyperintense line.	WD (tryptophan-aspartic acid) repeat domain 45	Member of the family of the WD40 proteins that promote protein-protein interactions and plays a role in autophagy, cell cycle and transcriptional control, and transduction. WDR45 is critical for autophagosome formation.
*FA2H* (611026)	Fatty acid hydroxylase-associated neurodegeneration (FANH) (612319)—AR Rare	Childhood-onset gait impairment caused by spastic paraplegia, ataxia and dystonia. Seizures and optic atrophy may be present. Hypointensity of the GP in some patients.	Fatty acid 2 hydroxylase	Enzyme that catalyzes the hydroxylation of fatty acids at position 2 of the N-acyl chain; 2-hydroxy-fatty acids are a precursor for ceramide synthesis (component of myelin sheaths).
*FTL* (134790)	Neuroferritinopathy (NF) (606159)—AD Rare	Adult-onset chorea with cognitive defects and psychiatric features. Cerebellar ataxia, parkinsonism, blepharospam and orolingual-mandibular dyskinesia. Hypointensity in basal ganglia, mainly GP and SN.	Ferritin	Major storage protein for cellular iron (up to 4500 iron atoms) consisting of a heavy chain with ferroxidase activity and a light chain that aids mineralization within the ferritin structure.
*CP* (117700)	Aceruloplasminemia (604290)—AR Rare	Adult-onset form with cognitive decline, cerebellar ataxia, and craniofacial dyskinesia, besides of retinal degeneration, and even, diabetes caused by the accumulation of iron. Hypointense striatum, thalamus and dentate.	Ceruloplasmin	Ferroxidase that facilitates ferroportin-mediated cellular iron export
*DCAF17* 612515	Woodhouse-Sakati syndrome (WSS) (241080)—AR Rare	Multisystem disorder that presents hypogonadism, hair thinning-to-alopecia, diabetes mellitus, deafness, intellectual disability, extrapyramidal features. Additional symptoms are seizures, polyneuropathy, thyroid dysfunction, keratoconus, and syndactyly. Hypointensity of the GP and SN.	Db1 and Cul4-associated factor 17	Unclear function. DCAF17 seems to be associated with protein ubiquitination involved in DNA damage and cell cycle control.
*ATP13A2* (610513)	Kufor-Rakeb syndrome (KRS) (606693)—AR Two probands	Onset is usually in adolescence. KRS is characterized by juvenile parkinsonism, dystonia, eye movement abnormalities, autonomic dysfunction, dementia, and psychiatric features, Hypointensity in the basal ganglia/caudo-putamen.	ATPase cation transporting 13A2	P-type ATPase associated with membranes of lysosomes, which is a divalent cation transporter that uses the energy stored in ATP to transport ions across membranes. Mislocalization of ATP13A2 to the endoplasmic reticulum and Zn^2+^ dyshomeostasis are crucial processes.
*COASY* (609855)	COASY protein-associated neurodegeneration (CoPAN) (615643)—AR Five probands	Early onset gait impairment and learning difficulties, with further dystonia, and spasticity. Hypointensity with central hyperintensity of the GP.	CoA synthase	Bifunctional enzyme that catalyzes the final two steps of the CoA synthesis.

OMIM: online mendelian inheritance in man, https://www.omim.org/; MIM*: gene; MIM#: phenotype; NBIA: neurodegeneration with brain iron accumulation; MIM: mendelian inheritance in man; AR: autosomal recessive; XD: X-linked dominant; AD: autosomal dominant; CoA: coenzyme A; GP: globus pallidus; SN: substantia nigra.

**Table 2:** NBIA-like forms.

Gene (OMIM*)	Disease (OMIM#) Inheritance	Clinical and MRI Findings	Protein	Function	References
*FBX07*	Parkinson disease 15 (260300) AR	A girl with a parkinsonian-pyramidal syndrome and brain iron accumulation. Light vermian atrophy progressing to cerebral and cerebellar atrophy, and a hot cross bun sign suggesting ponto-cerebellar tract degeneration. Excessive brain iron deposition in the GP and SN appeared as hypointense signal on iron-sensitive sequences	F-box only protein 7	Ubiquitin protein ligase involved in ubiquitin proteasome activity and parkin-mediated mitophagy.	[2]
*FUCA1* (612280)	Fucosidosis (230000) AR	A 14 year-old girl with progressive fixed dystonia and red spots on the skin. Axial T2-weighted images showed slightly hyperintense spot within a hypointense GP and mild hyperintensity of the subcortical white matter consistent with diffuse mild hypomyelination. Over the years, brain atrophy was noticed.	α-L-fucosidase	Lysosomal enzyme involved in the degradation of fucose-containing glycoproteins and glycolipids.	[3]
*GLB1* (611458)	GM1-gangliosidosis type I (infantile form; 230500) type II (juvenile form; 230600) type III (adult form; 8230650) AR	Two brothers with the juvenile form of GM1 gangliosidosis. A T2-weighted image revealed significant cerebral atrophy with barely noticeable hypointense signals in GP in the proband (9 years old). The cerebral atrophy and paramagnetic signals in the basal ganglia became more pronounced over the years. His affected brother presented a similar clinical course.	β-galactosidase-1	Lysosomal hydrolase that cleaves the terminal β-galactose from ganglioside substrates and other glycoconjugates.	[4]
		A 12 year-old boy with GM1 gangliosidosis type II. MRI revealed a hypointense T2 signal in the SN and in the GP, with marked hypointensity in susceptibility-weighted imaging and slight T2 hyperintensity in the posterior part of the putamen.			[5]
*GTPBP2* (607434)	Jaberi-Elahi syndrome (617988) AR	Three affected siblings who presented a complex neurologic disorder accompanied with mental deficiency and ataxia and dystonia features. Brain MRI showed cerebellar vermian atrophy, and susceptibility weighted imaging revealed hypointensity signal in the GP and SN suggesting NBIA.	GTP-binding protein 2	GTP-binding protein that belongs to the GTPase superfamily whose members play a role in a wide variety of biological process (cell proliferation and differentiation, intracellular transport, regulation of cytoskeleton, and protein synthesis).	[6]
*SPC2* (184755)	Leukoencephalopathy with dystonia and motor neuropathy (613724) AR	Adult-onset disease with brain MRI characteristic of NBIA: T2-weighted imaging showed signal change in the subcortical white matter, thalamus, globus pallidus cerebral peduncles, and pons. Susceptibility-weighted imaging showed signal dropout in the GP, SN, red nuclei, and dentate nuclei	Sterol carrier protein-2	Peroxisomal enzyme with thiolase activity that is required for the breakdown of branched-chain fatty acids.	[7]
*TBCE* (604934)	Encephalopathy with amyotrophy and optic atrophy (617207) AR	Patients suffering from an early-onset and progressive neurodegenerative encephalopathy with distal spinal muscular atrophy, and brain iron accumulation in the 2nd decade at the level of the GP and SN.	Tubulin folding cofactor E	Chaperone required for the proper tubulin folding and polymerization.	[8]
*KMT2B* (606834)	Dystonia 28, childhood-onset (617284) AD	Several patients suffering from an early-onset progressive dystonia, with subtle, low pallidal signal on T2*, diffusion and susceptibility weighted sequences, particularly affecting the lateral aspect of the GP externa.	Lysine-specific methyltransferase 2B	Histone lysine methyltransferase involved in methylation of histone H3 at lysine 4 (H3K4)	[9]
*DDHD1* (614603)	Spastic paraplegia 28 (609340) AR	A 55 year-old man with a complex form of spastic paraplegia with retinal dystrophy. MRI revealed a thin corpus callosum, a billateral T2 hyposignal of the internal pallidi and a hypersignal of the supratentorial white matter.	DDHD domain-containing protein 1	Phospholipase that hydrolyzes phosphatidic acid and attenuates cell activation	[10]
*AP4M1* (602296)	Spastic paraplegia 50 (612936) AR	Three affected relatives who exhibited very early psychomotor retardation with spasticity and severe mental deficiency. MRI showed global cerebral atrophy, white matter loss asymmetric ventriculomegaly and thining of the splenium of the corpus callosum. T2 sequences revealed symmetric mild hypointensity of the GP.	Adaptor-related protein complex 4 subunit MU-1	Subunit of the heterotetrameric adaptor protein (AP) complex, which plays a role in the recognition and sorting of cargo proteins with tyrosine-based motifs from the trans-golgi network to the endosomal-lysosomal system	[11]
*AP4S1* (607243)	Spastic paraplegia 52 (614067) AR	AP4 deficiency with ataxia and optic atrophy in a girl who presented with bilateral hypointense signal alterations in the GP in T2-weighted, suggesting abnormal iron accumulation.	Adaptor-related protein complex 4 σ-subunit MU-1	Subunit of the heterotetrameric adaptor protein (AP) complex, which plays a role in the recognition and sorting of cargo proteins with tyrosine-based motifs from the trans-golgi network to the endosomal-lysosomal system.	[12]
*RNASEH2B* (610326)	Aicardi-Goutieres syndrome 2 (610181) AR	Two affected siblings who manifested encephalopathy with spastic quadriplegia and anarthria but preserved intellect. MRI revealed hypointensity of GP on T2, consistent with iron deposition	Ribonuclease H2 subunit B	Subunit of the human ribonuclease H2 enzyme complex that cleaves ribonucleotides from RNA-DNA duplexes.	[13]
*LAMAN* (609458)	α-Mannosidosis types I and II (248500) AR	A 34 year-old man with juvenile onset of α-mannosidosis type II with cerebral and cerebellar atrophy and bilateral hypointensities in the thalamus, GP and putamen on T2-(turbo spin echo; TSE)-weighted images, suggestive of iron accumulation. The SN and red nucleus were also hypointense on T2-(TSE)-weighted images. The basal ganglia were isointense on T1-weighted images.	α-mannosidase	Lysosomal hydrolase that cleaves α-linked mannose residues from the nonreducting end of N-linked glycoproteins.	[14]
*REPS1* (614825)	NBIA 7 (617916) AR	Two sisters affected by an early-onset condition (trunk hypotonia, progressive cerebellar ataxia, pyramidal syndrome). MRI of the proband at age of 15 showed progressive cerebellar and cerebral atrophy, and hypointensity of the SN and GP consistent with iron deposition. Her affected sister at age of 2 showed no alterations.	RALBP1-associated Eps domain-containing protein 1	Protein involved in endocytosis and vesicular transport. REPS1 interacts with endocytic scaffold intersectin 1, an essential player in clathrin-mediated endocytosis.	[15]
*CRAT* (600184)	NBIA 8 (617917) AR	A girl suffering from an early-onset condition with slowly progressive spinocerebellar degeneration. MRI showed cerebellar atrophy and posterior leukodystrophy at 15.5 years old, besides of hypointensity of the SN and GP consistent with iron deposition.	Carnitine acetyltransferase	Enzyme involved in the control of the acyl-CoA/CoA ratio in mitochondria, peroxisomes, and endoplasmic reticulum.	[15]
*MANEAL* (NA) *OSTM1* (607649)	NA Osteopetrosis (259720) AR	Boy, carrier of homozygous mutations in *MANEAL* and *OSTM1*, with an early-onset complex neurodegenerative disorder. MRI demonstrated massive brain atrophy and symmetrical iron accumulation in GP, corpora mamillaria and cerebral peduncles, which may due to defects on *MANEAL.*	Mannosidase endo-α like protein Ostepetrosis-associated transmembrane protein 1	Unknown function/OSTM1 plays a role in subcellular endosome/lysosome dispersion and autophagy processes, which may be related to lysosomal dysfunction.	[16]
*MTSS1L* (616957)	NA AR	A 10 year-old girl with microcephaly, intellectual disability, developmental delay, and hypotonia. Brain MRI at 5 years of age was unremarkable, and when repeated at age of 10, showed iron accumulation in GP bilaterally. Her sister (7 years) is similarly affected; her MRI at age of 14 months did not show iron deposition.	Metastasis suppressor 1-like	Protein involved in plasma membrane dynamics that has a conserved N-terminal domain (IRSP53/MIM homology domain or an inverse BAR domain).	[17]
*VAC14* (604632)	Striatonigral degeneration childhood-onset (617054) AR	Two siblings affected by retinitis pigmentosa and an early childhood severe progressive spastic paraparesis with learning disabilities. MRI revealed at age of 9, decreased signal of the GP and SN (axial T2*) and striatal T2 prolongation (axial T2).	VAC14 or ArPIKfyve (associated regulator of the PIKfyve complex)	Component of the PIKfyve complex that tightly regulates the level of PtdIns(3,5)P2 in endosomal membranes.	[18]

OMIM: online mendelian inheritance in man, https://www.omim.org/; MIM*: gene; MIM#: phenotype; NBIA: neurodegeneration with brain iron accumulation; MIM: mendelian inheritance in man; AR: autosomal recessive; AD: autosomal dominant; MRI: magnetic resonance imaging; GP: globus pallidus; SN: substantia nigra.
